# Intratumoral administration of *Hibiscus sabdariffa-*derived anthocyanins exerts potent antitumor effects in murine cancer models

**DOI:** 10.3389/fimmu.2025.1549890

**Published:** 2025-03-07

**Authors:** Miriam Ezcurra-Hualde, Juan Florencio Gómez-Leyva, Efren Juarez-Curiel, Yanelli Jaquelinne Regalado-Noyola, Nuria Ardaiz, Noelia Casares, David Ruiz-Guillamon, Silvia Monserrat Rodríguez-Leon, Flor Yohana Flores-Hernández, Leire Arrizabalaga, Aline Risson, Román García-Fuentes, Celia Gomar, Virginia Belsue, Fernando Aranda, Pedro Berraondo, Maritza R. Garcia-Garcia

**Affiliations:** ^1^ Program of Immunology and Immunotherapy, Cima Universidad de Navarra, Cancer Center Clínica Universidad de Navarra (CCUN), Pamplona, Spain; ^2^ Navarra Institute for Health Research (IDISNA), Pamplona, Spain; ^3^ Laboratorio de Biología Molecular, TecNM-Instituto Tecnológico de Tlajomulco, Tlajomulco de Zúñiga, Jalisco, Mexico; ^4^ Solid Tumor Program, Cima Universidad de Navarra, Cancer, Center Clínica Universidad de Navarra (CCUN), Pamplona, Spain; ^5^ Escuela de Nutrición, Universidad Autónoma de Guadalajara, Zapopan, Jalisco, Mexico; ^6^ Unidad de Biotecnología Médica y Farmacéutica, Centro de Investigación y Asistencia en Tecnología y Diseño del Estado de Jalisco, Guadalajara, Mexico; ^7^ Spanish Center for Biomedical Research Network in Oncology (CIBERONC), Madrid, Spain

**Keywords:** *Hibiscus sabdariffa L*., anthocyanins, intratumoral therapy, antitumor activity, immune modulation

## Abstract

**Introduction:**

Cancer remains the leading cause of death worldwide, with increasing incidence rates. Natural compounds have gained attention as potential therapeutic agents due to their bioactive properties. Anthocyanins, particularly delphinidin-3-sambubioside (Dp-3-sam) and cyanidin-3-sambubioside (Cn-3-sam), are flavonoids with antioxidant and potential antitumor properties. This study investigates the antitumor effects of anthocyanins extracted from *Hibiscus sabdariffa* L. (*H. sabdariffa*), administered intratumorally, and their potential as adjuvants to chemotherapy.

**Methods:**

Anthocyanins were extracted from *H. sabdariffa* and characterized using high-performance liquid chromatography (HPLC). The total phenolic content was determined using the Folin–Ciocalteu method. Antioxidant activity was assessed through DPPH, ABTS, and FRAP assays. The antiproliferative effects of Dp-3-sam and Cn-3-sam were evaluated in vitro using MCA-205 fibrosarcoma and CT26 colon carcinoma cell lines. In vivo studies were conducted on mouse tumor models to assess tumor growth inhibition following intratumoral administration of anthocyanins alone or in combination with doxorubicin. The impact on angiogenesis, immune cell recruitment, and long-term immune memory was also analyzed.

**Results:**

HPLC analysis confirmed the presence of Dp-3-sam and Cn-3-sam in the *H. sabdariffa* extract. The anthocyanins exhibited significant antioxidant activity in all assays. In vitro studies demonstrated dose-dependent inhibition of cancer cell proliferation. In vivo, intratumoral administration of anthocyanins led to a significant reduction in tumor growth. The combination of anthocyanins with doxorubicin further enhanced tumor suppression. Mechanistically, Dp-3-sam and Cn-3-sam reduced angiogenesis and promoted immune cell recruitment but did not elicit an effective antitumor immune response alone. However, co-administration with doxorubicin reversed this limitation, leading to increased immune activation and resistance to tumor rechallenge, suggesting the induction of long-term immune memory.

**Discussion:**

These findings highlight the potential of *H. sabdariffa*-derived anthocyanins as adjuvants in cancer therapy. When administered intratumorally, they enhance chemotherapy efficacy and immunogenicity. However, further studies are needed to optimize dosing strategies, evaluate long-term safety, and assess clinical applicability.

## 1 Introduction

Cancer remains one of the leading causes of death worldwide, with a rapidly increasing incidence (1). In the field of oncology therapy, phytomedicine has been historically relevant. Some plant-derived compounds with clinical relevance in cancer management have been isolated in the past (e.g., taxol, vincristine, and vinblastine) (2). Therefore, the use of combined chemotherapy with traditional herbal medicine as a chemotherapeutic adjuvant has been widely embraced as a potential strategy to fight cancer. While phytochemicals or standardized herbal extracts are not free of the risk of potential adverse effects, in cancer research, previous evidence suggests that combining chemotherapy with specific plant-derived compounds offers several benefits. These advantages could include synergistic induction of specific immune responses against tumors, increased tumor sensitivity to chemotherapy, and mitigation of chemotherapy-induced toxicity (3, 4).

*Hibiscus sabdariffa Linn* (*H. sabdariffa*), a member of the Malvaceae family, is widely known as roselle, red sorrel, Florida cranberry, or Jamaica flower, depending on the region. Native to Africa, it is now cultivated globally, particularly in tropical and subtropical areas (5). It is highly valued for its culinary, cosmetic, and medicinal applications (6). Traditionally, *H. sabdariffa* has been used across Mexico, China, Egypt, India, South America, and other regions as an adjuvant herbal-derived product to improve health in patients with conditions such as hypertension, hyperlipidemia, diabetes mellitus, obesity, coronary disease, and even cancer (7). In terms of its anticancer activity, several potential antiproliferative, cytotoxic, antimetastatic, and proapoptotic effects have been attributed to *H. sabdariffa* in both *in vitro* and *in vivo* models of T-lymphoblastic leukemia, melanoma, and breast, prostate, and gastric cancer (8, 9).

The anticancer potential of *H. sabdariffa* is believed to be due to its nutritional and phytochemical properties. Its constituents include a broad spectrum of organic acids, sugars, fats, proteins, minerals, vitamins, and phenolic compounds (10). Specifically, among all its bioactive compounds, the anthocyanins—which are prominent water-soluble polyphenols contained in the calyces of *H. sabdariffa* that impart a deep red color pigment—have garnered significant scientific interest because of their bioactive properties and potential health benefits (7). Notably, delphinidin-3-sambubioside (Dp3-sam) and cyanidin-3-sambubioside (Cn3-sam), the most abundant anthocyanins in *H. sabdariffa*, have been extensively studied for their antioxidant, antibacterial, antiviral, anthelmintic, and anticancer activities (11). In particular, Dp-3-sam has been implicated in apoptosis induction via an ROS-mediated mitochondrial dysfunction pathway in human leukemia cells (12), and it has even been patented for treating malignant melanoma (13). In addition, in the cancer field, anthocyanins have been implicated in the inhibition of cell proliferation, invasion, migration, angiogenesis, and inflammation (14).

In this study, we administered *H. sabdariffa* and its derived anthocyanins (Dp-3-sam and Cn3-sam) via the intratumoral route with the aim of investigating their antitumor potential. In addition, a combination therapy composed of anthocyanins with doxorubicin was evaluated in carcinoma mouse models to promote an effective immune response. This work explores their potential as adjuvant phytochemicals for cancer therapy.

## 2 Materials and methods

### 2.1 Plant collection, processing and characterization

Plants from *Hibiscus sabdariffa L.* var. Americana were collected and authenticated at the herbarium of the Technological Institute of Tlajomulco (ITTJ-7101) in Jalisco, Mexico. The anthocyanins were extracted from the dried calyx using an acidified 70% ethanol solution. For the purification of anthocyanins (Dp3-sam/Cn3-sam), the crude extract was applied to a column with Amberllite^®^ XAD-7HP resin (Sigma−Aldrich) previously activated with 0.1% HCl. Then, the crude anthocyanin extract was loaded and eluted with 96% ethanol acidified with 0.1% HCl at a flow rate of 1 mL/min. The extract obtained was evaporated, lyophilized and stored at -20°C until use. High-performance liquid chromatography with a diode array detector or electrospray-quadrupole-time-of-flight tandem mass spectrometry detector (HPLC−DAD−ESI−TOF) analysis, total phenolic content (TPC) determination, and analysis of antioxidant activity via DPPH (2,2-diphenyl-1-picrylhydrazyl), ABTS (2,2′-azino-bis(3-ethylbenzothiazoline-6-sulfonic acid)), and FRAP (ferric ion reducing antioxidant potential) assays were subsequently conducted according to the methods of (10). Both the H. sabdariffa and the anthocyanin extracts were lyophilized and stored at -20°C before use. Freshly extracted extracts were prepared and protected in the dark at the time of use. For *in vitro* experiments, the extracts were dissolved in sterile cell culture media containing 0.02% dimethyl sulfoxide (DMSO) as an extraction vehicle, whereas for *in vivo* tests, the extracts were easily dissolved in phosphate buffer solution (PBS) and prepared under sterile conditions.

### 2.2 Mice and cell lines

All animal experiments were approved by the Institutional Ethics Committee of the Universidad de Navarra (protocol number 081-22). Female C57BL/6 and BALB/c mice (5–7 weeks old) were purchased from Envigo (Huntingdon, UK) and housed under pathogen-free conditions at the Center for Applied Medical Research (CIMA, Universidad de Navarra) animal facility. Mycoplasma-free MCA-205 and CT26 cell lines for fibrosarcoma and colon carcinoma, respectively, were provided by Dr. Ignacio Melero (CIMA, Universidad de Navarra) and were cultured in RPMI-1640 Glutamax medium supplemented with 10% fetal bovine serum, 1% L-glutamine, and 1% penicillin/streptomycin (Gibco, Thermo Fisher Scientific, Spain). The cells were maintained at 37°C with 5% CO_2_ and 95% humidity. Once the cells reached 80% confluence, they were detached with 0.25% trypsin/EDTA, stained with trypan blue, and counted in a Neubauer chamber.

### 2.3 *In vitro* cytotoxic and antiproliferative effects

The cytotoxic effects of different concentrations of *H. sabdariffa* and Dp3-sam and Cn3-sam extracts (from 0.05 to 10.0 mg/mL) were assayed in MCA-205 and CT26 cells after 24 and 48 h via the [^3^H]thymidine incorporation method presented by (15). Cytotoxicity and proliferation were also evaluated via an xCELLigence Real‐Time Cell Analysis Instrument (RTCA) (ACEA Biosciences). MCA-205 and CT26 cells (3.5 × 10^4^ cells/well) were plated in a 16-well plate (Aligent, Adelaida, South Australia, AUS). Then, the plates were placed into the RTCA equipment, and baseline impedance measurements were taken for 6 h. After this time, different concentrations of *H. sabdariffa* and Dp3-sam and Cn3-sam extracts or doxorubicin (25 µM) were added to the wells, and impedance measurements were recorded every 5 min for 48 h. RTCA software was used for the analysis, and the cell index curve was normalized to the time point at which the cells were treated with the extracts or the doxorubicin control.

### 2.4 *In vivo* antitumor effects

Seven- to eight-week-old C57BL/6 and BALB/c mice were subcutaneously implanted into the right flank with 5 x 10^5^ MCA-205 and CT26 tumor cells, respectively. Five days postimplantation, the tumors were measured with calipers, and the tumors were collected from the mice in the different treatment groups to ensure similar tumor sizes in all the groups. For the *H. Sabdariffa-* or Dp3-sam/Cn3-sam-treated groups, sterile extracts were administered through direct intratumoral injection at different times (multidoses) at days 5, 12, and 19 after tumor implantation with 50 μL at a concentration of 500 mg/kg of whole-body weight diluted in PBS. To promote an effective immune response, in combination with Dp3-sam/Cn3-sam anthocyanins, 20 µl of a 10 mM solution of doxorubicin diluted in PBS was subsequently administered on day 5 (16), and the mice subsequently received Dp3-sam/Cn3-sam on days 6, 12, and 19 postintratumoral injection. The control group received the same volume of PBS. The first treatment was administered at a single central site within the tumor, followed by subsequent doses at multiple points where tumor cells began to grow. A rechallenge of the same cells was subsequently performed on fully cured mice.

### 2.5 RNA extraction and sequencing

To investigate the underlying mechanisms of the antitumor activity of anthocyanins, RNA sequencing (RNAseq) studies were performed. For *in vitro* RNA-seq, MCA-205 cells (5 × 10^5^ cells) were treated with Dp3-sam/Cn3-sam for 6 h before RNA isolation. For the *in vivo* RNA-seq study, a subcutaneous MCA-205 mouse cancer model was used. After 10 days of tumor implantation, sterile Dp3-sam/Cn3-sam extract was administered via direct intratumoral injection. At 6 h after the mice received the intratumoral injection, they were euthanized, and the tumors were resected. Total RNA was isolated with an RNeasy Micro Kit (Qiagen, Hilden, ALE) and quantified with a NanoDrop spectrophotometer (Thermo Scientific). The RNA was subjected to quantity and quality control using a Qubit HS RNA Assay Kit (Thermo Fisher Scientific) and a 4200 TapeStation with High Sensitivity RNA ScreenTape (Agilent Technologies). RNAseq library preparation was performed using 100 ng of high-quality RNA (RIN > 7) and the Illumina Stranded mRNA Prep Ligation Kit following the manufacturer’s protocol. Libraries were sequenced via a NextSeq2000 (Illumina), and 40 million paired-end reads (Rd1/Rd2:51 bp) were sequenced for each sample.

### 2.6 Histological analysis

To assess the presence of epithelial cells and quantify neoangiogenesis in MCA-205 tumors treated with Dp3-sam/Cn3-sam, an immunohistochemistry (IHC) assay was performed to identify CD31 and CD3 markers. After 6 h, the mice that received Dp3-sam/Cn3-sam intratumorally were euthanized, and the tumors were resected. Then, the tumors were fixed in formalin, embedded in paraffin, and sectioned (3 µm). Hematoxylin and eosin (H&E) staining was used for morphology evaluation. Immunohistochemistry was performed with rat anti-mouse CD31 (1:400, clone SZ31, Dianova) and rabbit anti-CD3 (1:300, clone SP7, Thermo Scientific) antibodies. Antigen retrieval was achieved in Tris-EDTA buffer (pH 9) at 95°C for 30 minutes. The sections were incubated with primary antibodies overnight at 4°C, followed by incubation with secondary antibodies. Peroxidase activity was revealed with DAB+ (Agilent, Technologies, Adelaida, South Australia, AUS), and the sections were counterstained with hematoxylin, dehydrated, and mounted.

### 2.7 Statistical analysis

Statistical differences in multiple comparisons were analyzed with one-way ANOVA or the Kruskal−Wallis test and Sidak’s multiple comparisons test. Differences in tumor volume curves were determined using nonlinear fit tests. Survival differences were analyzed with the Mantel‐Cox test. GraphPad Prism version 8.0.2 (San Diego, California, USA) was used to analyze the data. Significant differences were set at p <0.05.

## 3 Results

### 3.1 Characterization and quantification of anthocyanins in *H. Sabdariffa*

**Figure 1:**
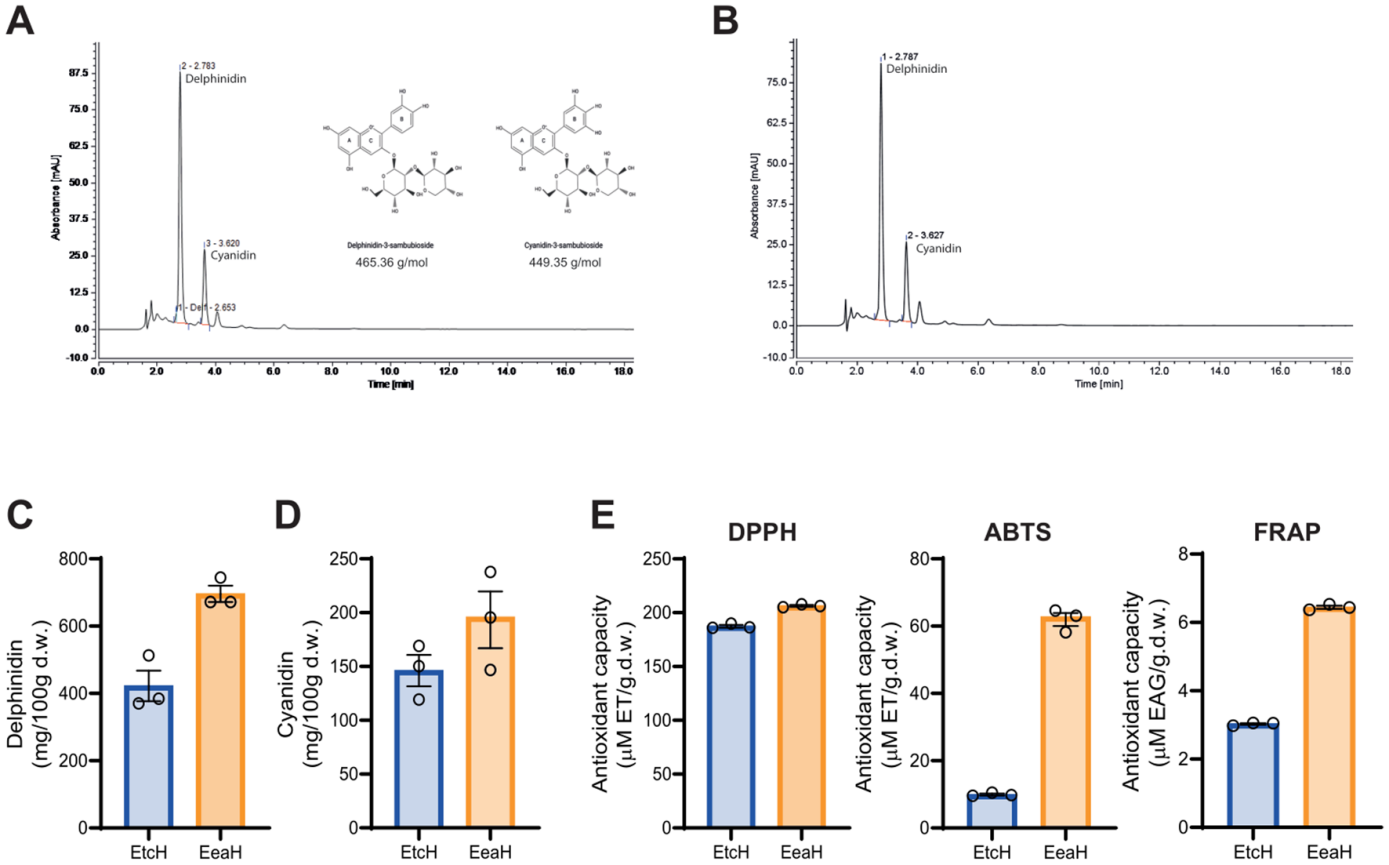
Anthocyanin characterization and antioxidant activity of *Hibiscus sabdariffa L.* extracts. **(A)** RP-HPLC chromatogram of *H. Sabdariffa* showing anthocyanidin peaks for delphinidin-3-sambubioside (Dp3-sam) and cyanidin-3-sambubioside (Cn3-sam). **(B)** RP-HPLC chromatogram of purified anthocyanins after XAD-7HP column chromatography. **(C, D)** Quantification of delphinidin and cyanidin in the ethanolic extract (EtcH) and purified extract (EeaH) via RP-HPLC. **(E)** Antioxidant activity of ethanolic (EtcH) and purified extracts (EeaH) determined via DPPH, ABTS, and FRAP assays. Significant differences in antioxidant capacity were noted between the extracts. The data are expressed as the means ± SDs.

The anthocyanin content of *H. sabdariffa* extract was determined via HPLC-DAD-ESI-TOF analysis, which identified two primary anthocyanins: Dp-3-sam and Cn3-sam. The chromatogram in **Figure 1A** displays clear peaks for both anthocyanins, confirmed by their retention times compared with those of standards. Further purification of these anthocyanins via Amberlite XAD-7 column chromatography (**Figure 1B**) resulted in enriched extracts of Dp3-sam and Cn3-sam, which were then quantified. **Figures 1C, D** show the concentrations of Dp3-sam and Cn3-sam in *H. sabdariffa* and the purified Dp3-sam and Cn3-sam extracts. The purified extract contained high concentrations of both anthocyanins (ranging from 708 to 216 mg/100 g d. w. with a ratio of delphinidin:cyanidin of 3:1). The quantity of extractable polyphenols (as TPC) in the dried extract samples was determined via the Folin–Ciocalteu method. The TPC of the purified anthocyanin extract was greater than that of the entire *H. sabdariffa* extract (46.26 ± 0.97 *vs* 27.83 ± 0.30 mg GAE), which is in accordance with the amount of anthocyanins in the purified Dp3-sam and Cn3-sam extracts (**Figure 1E**).

The antioxidant activity of the extracts was assessed via DPPH, ABTS, and FRAP assays. **Figure 1E** presents the antioxidant capacity of both the *H. sabdariffa* and the purified Dp3-sam and Cn3-sam extracts across the three assays. Compared with the entire *H. sabdariffa* extract, the purified extract exhibited greater antioxidant activity in all tests, with significant differences observed in the DPPH, ABTS, and FRAP assays (**Figure 1E**). These results indicate that the purified anthocyanin-enriched extracts possess potent free radical scavenging properties, which are directly associated with Dp3-sam and Cn3-sam, without the presence of organic acids or other components.

### 3.2 *In vitro* antiproliferative effects on cancer cell lines

**Figure 2:**
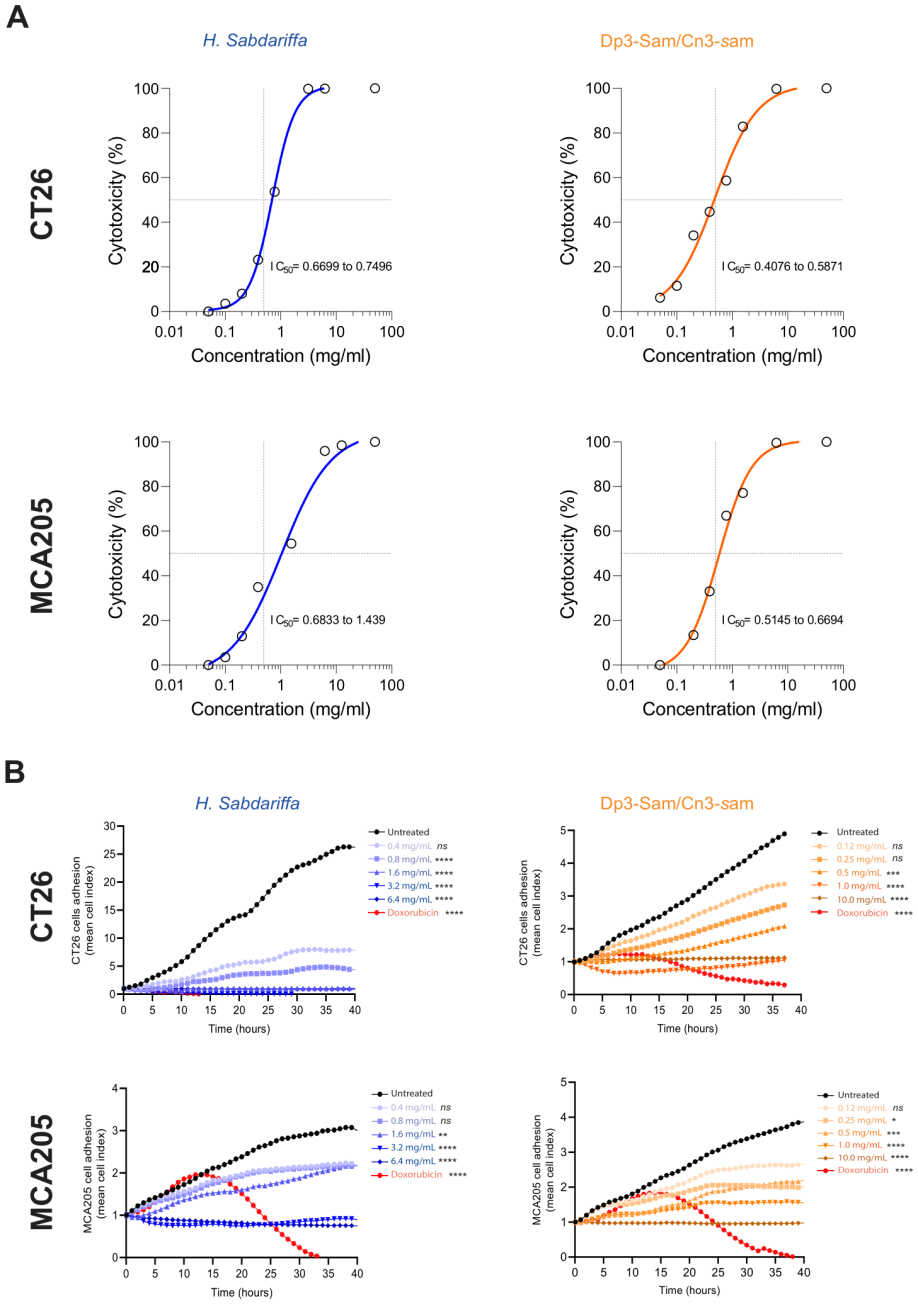
Antiproliferative effects of *Hibiscus sabdariffa L.* extracts on MCA-205 and CT26 cancer cells. **(A)** Thymidine incorporation assay in MCA-205 fibrosarcoma and CT26 colon carcinoma cell lines. A total of 1 × 10^5^ cells were incubated with various concentrations of anthocyanins for 24 and 48 hours. **(B)** Real-time analysis of cell adhesion via xCELLigence of 3.5 × 10^4^ MCA-205 and CT26 cells with different concentrations of *H. Sabdariffa* anthocyanins and doxorubicin. The data were analyzed via one-way ANOVA with the Kruskal−Wallis test. *p ≤ 0.05, **p ≤ 0.01, ***p ≤ 0.001, ****p ≤ 0.0001, ns, nonsignificant.

To evaluate the effects of *H. sabdariffa* and purified anthocyanin (Dp3-sam and Cn3-sam) extracts on the proliferation of the MCA-205 fibrosarcoma and CT26 colon carcinoma cell lines, the cells were treated for 24 h and 48 h with different concentrations of the indicated extracts. To monitor cell proliferation, the metabolic incorporation of 3H-thymidine into the cellular DNA was analyzed. As shown in **Figure 2A**, both the *H. sabdariffa* and the purified anthocyanin extracts significantly inhibited cell proliferation in a dose- and time-dependent manner. In addition, lower IC_50_ values were observed with anthocyanins than with the total *H. sabdariffa* extract, which indicates greater anthocyanin antiproliferative efficacy in MCA-205 and CT26 cells (**Figure 2A**). To confirm these findings, cytotoxicity was tested via real-time cell analysis via xCELLigence, which revealed noticeable cytotoxicity in cells treated with anthocyanins in comparison with those exposed to total *H. sabdariffa* extract, indicating greater efficacy of Dp3-sam and Cn3-sam for the disruption of cancer cell growth (**Figure 2B**).

### 3.3 Modulation of gene expression in MCA-205 cells treated with anthocyanins

**Figure 3:**
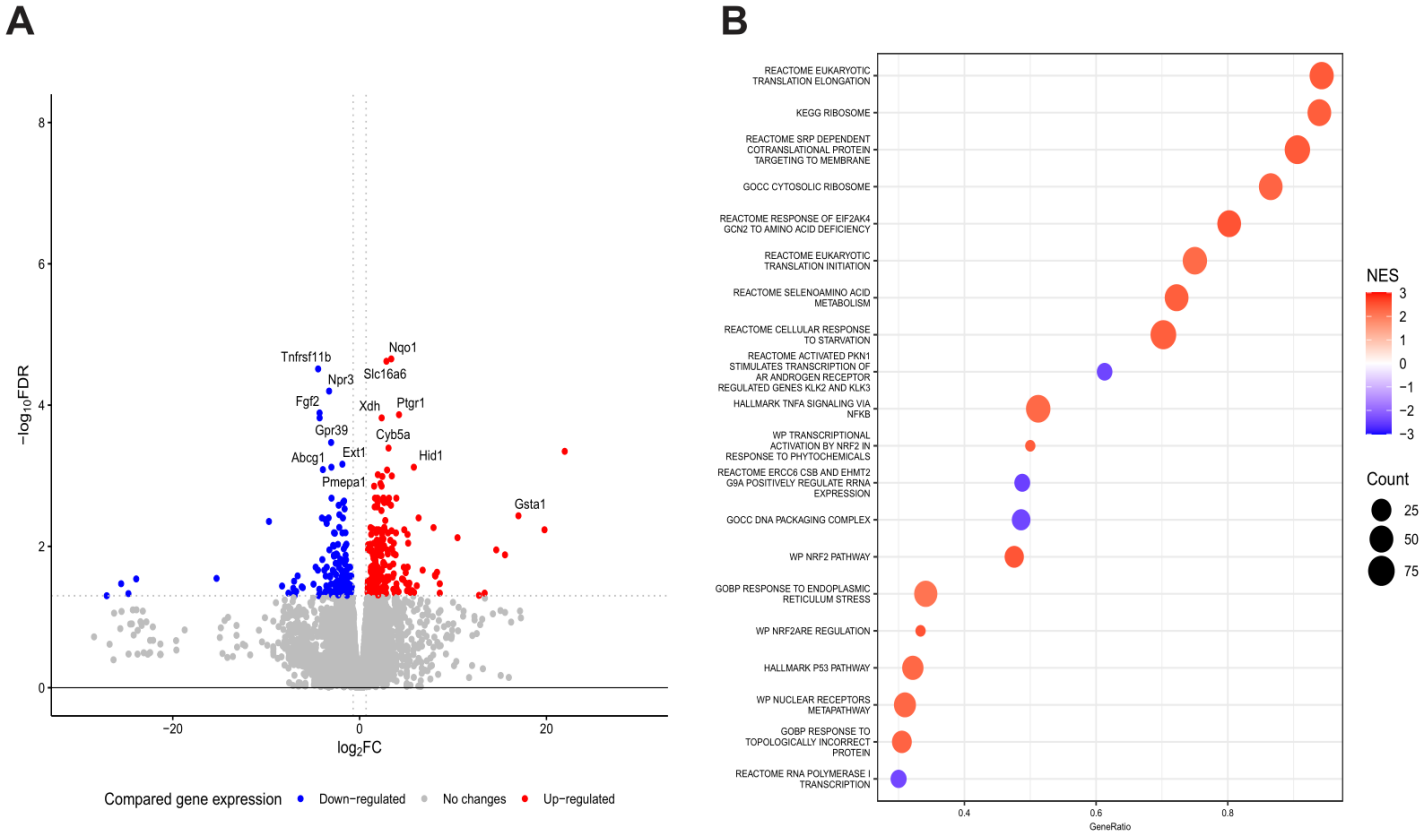
Gene expression analysis of the tumor response to anthocyanin treatment. RNA sequencing analysis in which 5 x 10^5^ MCA-205 cells were stimulated with Dp3-sam/Cn3-sam and analyzed vs. control cells. **(A)** Volcano plot showing DEGs in MCA-205 tumors treated with anthocyanins. **(B)** Gene set enrichment analysis (GSEA) identifying key pathways involved in the immune response, oxidative stress, and apoptosis.

Next, because Dp3-sam and Cn3-sam exhibited noticeable anticancer potential, RNA sequencing analysis was conducted to evaluate the modulation of gene expression in MCA-205 cells treated with Dp3-sam and Cn3-sam anthocyanins. The results revealed significant changes in gene expression following treatment (**Figure 3A**). Pathway enrichment analysis of modulated genes in MCA-205 cancer cells following treatment revealed significant changes in molecular processes related to protein synthesis, the stress response, cellular adaptation and survival (**Figure 3B**).

With respect to the upregulation of pathways involved in protein synthesis, we found robust activation of the cellular machinery responsible for the initiation and elongation of eukaryotic translation and ribosome biogenesis. Moreover, the upregulation of the cotranslational signal recognition particle (SRP), which is involved in the transport of proteins to the plasma membrane or the endoplasmic reticulum, supports the notion of enhanced synthesis and trafficking of secretory and membrane proteins, suggesting an adaptive response to treatment-induced stress.

On the other hand, MCA-205 cells also exhibited significant upregulation of eukaryotic translation initiation factor 2 alpha kinase 4 (EIF2AK4), which encodes the serine/threonine kinase GCN2, a key regulator of the integrated stress response that responds to amino acid depletion by monitoring the efficiency of protein synthesis. These findings suggest that cells sense nutrient scarcity and activate mechanisms to conserve resources while selectively synthesizing stress-related proteins. In addition, the response to the endoplasmic reticulum (ER) stress pathway was upregulated, reflecting the activation of the unfolded protein response aimed at restoring protein homeostasis under conditions of ER stress.

In addition, the modulation of gene expression in MCA-205 cells treated with anthocyanins revealed an effort to counteract the harmful effects of treatment, maintain homeostasis, and promote survival under therapeutic stress. In this context, a pronounced increase in antioxidant defenses was observed through the activation of the NF-E2-related factor 2 (NRF2) signaling pathway and the upregulation of the selenium amino acid metabolism pathway to counteract the oxidative stress caused by the treatment. Similarly, genes belonging to the TNF and NF-κB families were upregulated to modulate inflammation, cell survival, and resistance to apoptosis. Although the p53 pathway was also upregulated, MCA-205 cell gene expression exhibited metabolic adaptation for survival due to the upregulation of starvation pathways.

### 3.4 *In vivo* antitumor effects of anthocyanins in mouse models

**Figure 4:**
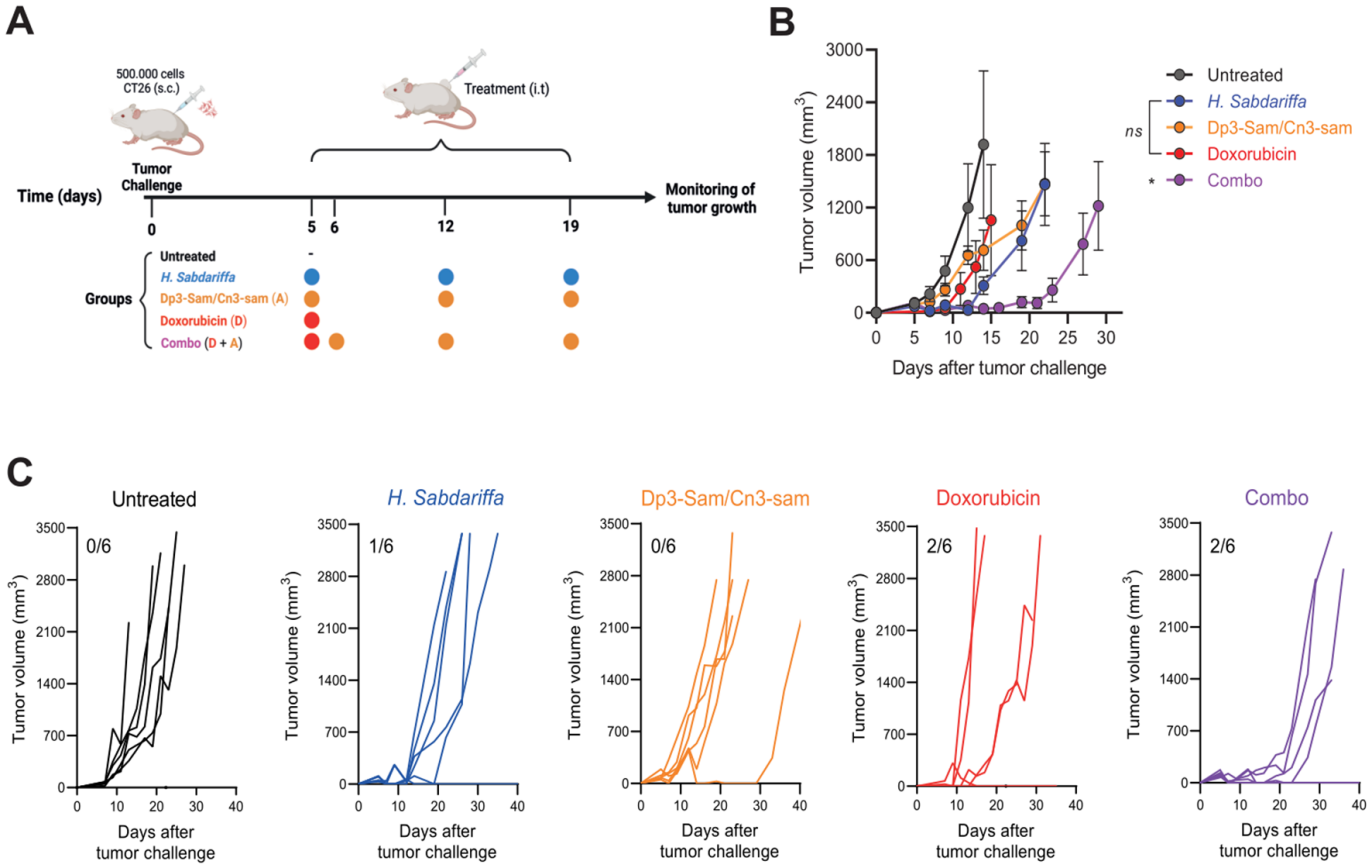
*In vivo* antitumor effects of *Hibiscus sabdariffa L*. extracts on the CT26 tumor model. **(A)** Graphical abstract of the assay. A total of 5 × 10^5^ CT26 cells were injected into the mice, which were then intratumorally injected with Dp3-sam/Cn3-sam, *H. Sabdariffa*, doxorubicin, or the combination. **(B)** Tumor volume measurements in BALB/c mice bearing CT26 colon carcinoma after treatment with *H. Sabdariffa*, Dp3-sam/Cn3-sam, doxorubicin and combo. **(C)** Tumor growth curves showing individual responses in each treatment group, including the number of fully cured mice. The data were analyzed via one-way ANOVA with Sidak’s multiple comparisons test. *p ≤ 0.05, ns, non-significant.

To evaluate the therapeutic efficacy of *H. Sabdariffa-*derived Dp3-sam and Cn3-sam anthocyanins, CT26 colon carcinoma-bearing mice were treated with intratumoral administration of the extracts (**Figure 4A**). Interestingly, injection of either *H. sabdariffa* extract or purified anthocyanins resulted in rapid delayed tumor growth, with a significant reduction in tumor mass observed within the first 24 h after treatment. However, tumor regrowth occurred from the tumor margins, indicating incomplete tumor eradication (**Figures 4B, C**). Consequently, the overall long-term effects on tumor dynamics are limited. Treatment with doxorubicin alone also modestly delayed tumor growth, but all the mice eventually developed subcutaneous tumors and were sacrificed at the end of the experiment (**Figures 4B, C**). Notably, the combination of doxorubicin with anthocyanins produced a synergistic antitumor effect, leading to a more substantial reduction in tumor size and a significant improvement in survival rates compared with either treatment alone (**Figures 4B, C**).

**Figure 5:**
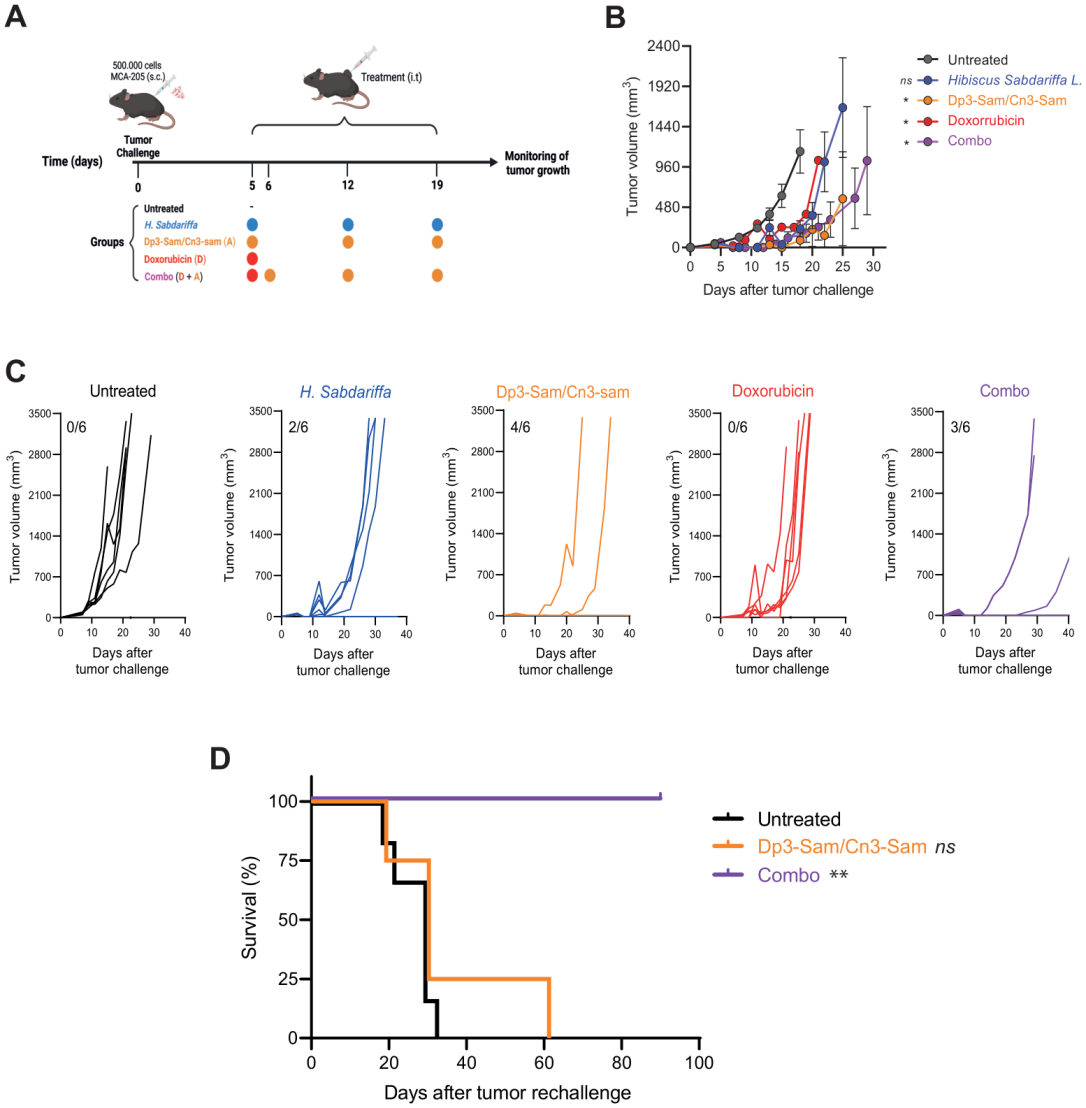
*In vivo* antitumor effects of *Hibiscus sabdariffa L*. extracts on MCA-205 tumor model. **(A)** Graphical abstract showing 5 × 10^5^ MCA-205 cells inoculated to mice and intratumorally injected with *H. Sabdariffa*, Dp3-sam/Cn3-sam, doxorubicin or the combination. **(B)** Tumor volume measurements in C57BL/6 mice bearing MCA-205 fibrosarcomas after treatment with *H-Sabdariffa*, Dp3-sam/Cn3-sam, doxorubicin and combo. **(C)** Tumor growth curves showing individual responses in each treatment group, including the number of fully cured mice. **(D)** Mice previously cured of MCA-205 tumors were rechallenged with the same tumor cells to assess the long-term immune response. Tumor volume over time was measured in previously treated and naïve control mice. Kaplan−Meier curve of mouse survival. The data were analyzed via one-way ANOVA with Sidak’s multiple comparisons test and the log rank test. *p ≤ 0.05, **p ≤ 0.01, ns, non-significant.

To further validate these findings, MCA-205-derived fibrosarcoma tumors were treated intratumorally with purified anthocyanins (**Figure 5A**). In this model, treatment with anthocyanins alone successfully eradicated tumors in 4 out of 6 mice. However, unlike in the CT26 model, the addition of doxorubicin did not improve the therapeutic outcome. However, an interesting effect was observed when the immune memory response was evaluated (**Figures 5B, C**).

To assess the long-term immunogenic effects of the therapy, mice that were cured of tumors following treatment with anthocyanins, with or without doxorubicin, were rechallenged with tumor cells. In previously cured mice treated with anthocyanins alone, the rechallenged tumors grew at a similar rate to those in untreated naïve mice. In contrast, the mice that rejected the tumor after combined treatment with anthocyanins and doxorubicin exhibited significantly slower tumor growth upon rechallenge, suggesting that the combination therapy induced a robust immune memory response (**Figure 5D**).

### 3.5 Mechanism of the therapeutic effect of anthocyanins

**Figure 6:**
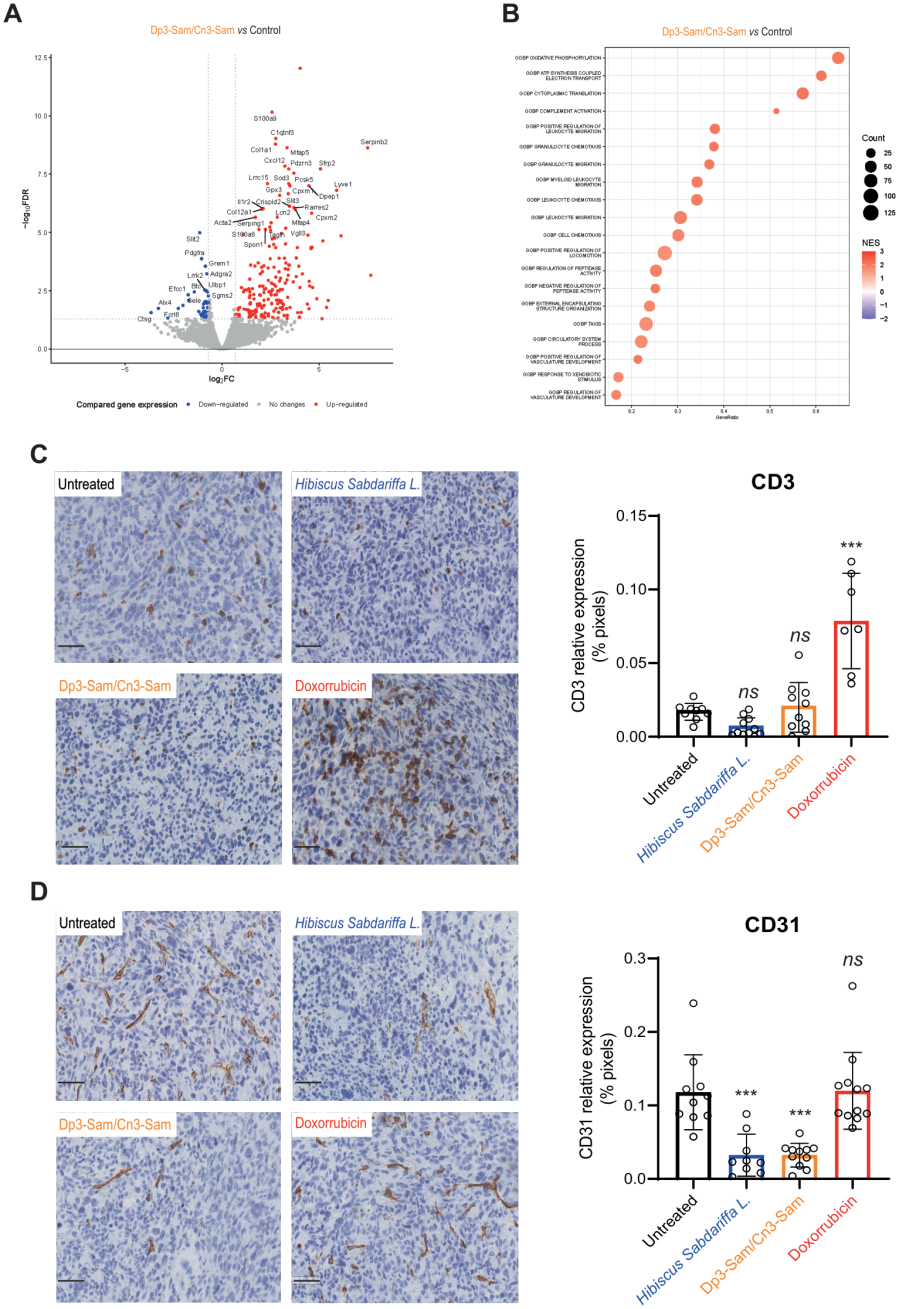
Mechanism of action of anthocyanin treatment in the MCA-205 tumor model. Ten days after tumor implantation, sterile Dp3-sam/Cn3-sam extract was administered intratumorally. Mice were euthanized 6 hours after injection, and tumors were resected for analysis. **(A)** Volcano plot showing the upregulated and downregulated genes in tumors treated with purified anthocyanins. **(B)** GSEA plot illustrating significant pathway activation following anthocyanin treatment, highlighting oxidative stress and immune-related pathways. **(C)** Representative images and quantification of CD3^+^ T-cell infiltration in tumors treated with *H. Sabdariffa* anthocyanins. **(D)** CD31^+^ endothelial cell density, showing reduced vascularization in treated tumors. The data were analyzed via one-way ANOVA with Tukey’s multiple comparisons test. ***p ≤ 0.001, ns, non-significant.

To investigate the underlying mechanisms of the therapeutic activity of anthocyanins *in vivo*, we performed gene expression analysis via RNA-seq. **Figure 6A** presents a volcano plot showing significantly upregulated and downregulated genes in MCA-205 tumors treated with purified anthocyanins. Gene set enrichment analysis (GSEA) identified key pathways involved in immune response activation, oxidative stress, and vascular development (**Figure 6B**). Treatment with anthocyanins significantly modulated inflammation-related pathways. Specifically, pathways involved in complement activation, leukocyte migration and chemotaxis, granulocyte migration, neutrophil migration, and myeloid leukocyte migration were upregulated, supporting the recruitment of innate immune cells. Interestingly, there was an increase in the regulation of vasculature development, which may facilitate immune infiltration and suggest a regulatory feedback mechanism in response to vascular destruction.

To validate these findings, we performed immunohistochemical detection of mouse CD3 and CD31 markers in treated tumors. Our data indicate that doxorubicin treatment increases the infiltration of CD3^+^ T cells, as expected, due to its capacity to induce immunogenic cell death. In contrast, despite their rapid antitumor effects, total *H. sabdariffa* extracts and purified Dp3-sam and Cn3-sam anthocyanins failed to promote the recruitment of T lymphocytes (**Figure 6C**). Regarding CD31^+^ endothelial cells, doxorubicin had no effect, whereas the extracts reduced the number of vascular cells in tumors compared with controls (**Figure 6D**).

## 4 Discussion

The findings of this study provide important insights into the therapeutic potential of *H. Sabdariffa-*derived Dp3-sam and Cn3-sam anthocyanins in cancer treatment, particularly their ability to modulate the tumor microenvironment and suppress tumor growth. Our results demonstrated that Dp3-sam and Cn3-sam, the primary anthocyanins isolated from *H. Sabdariffa*, exhibit potent antioxidant properties and significant antiproliferative effects on both fibrosarcoma (MCA-205) and colon carcinoma (CT26) cell lines. These findings align with previous reports of the antioxidant and anticancer properties of anthocyanins, underscoring their therapeutic potential as both free radical scavengers and modulators of tumor biology (17).

Importantly, to the best of our knowledge, no previous *in vivo* study has considered the intratumoral administration of Dp3-sam and Cn3-sam anthocyanins to evaluate their therapeutic efficacy. In this work, according to our findings, the intratumoral administration of anthocyanin extracts led to a rapid reduction in the tumor mass, although regrowth from tumor margins suggests that single-agent therapy may not be sufficient for complete tumor eradication. The observed synergistic effects of combining anthocyanins with doxorubicin, particularly in terms of long-term survival and immune memory, highlight the potential of this combination strategy in improving treatment efficacy. These results suggest that anthocyanins can enhance the effects of traditional chemotherapeutics by modulating the tumor immune landscape and promoting innate immune cell infiltration.

Mechanistically, gene expression analysis revealed a complex tumor response to anthocyanin treatment. Notably, pathways involved in protein synthesis and the stress response were upregulated (18). These changes suggest that cancer cells adapt to treatment-induced stress by activating protective mechanisms that help maintain cellular homeostasis. While this may represent a compensatory survival strategy, it also suggests potential therapeutic targets for disrupting these adaptive processes and enhancing the efficacy of anthocyanin-based therapies.

Interestingly, although several immune-related pathways, including those involved in complement activation and innate immune cell migration, were upregulated following anthocyanin treatment, key cytotoxic immune cells such as CD3^+^ T cells were not concurrently upregulated. These findings indicate that while anthocyanins promote innate immune cell recruitment, they may impair the recruitment of cytotoxic cells with the ability to kill tumor cells directly. Immunohistological analysis supported this finding, revealing increased infiltration of CD3^+^ T cells following doxorubicin treatment but not anthocyanin treatment. This lack of T-cell recruitment indicates that anthocyanins alone may not elicit a strong adaptive immune response, which may account for the incomplete tumor eradication observed *in vivo*. Future studies should explore strategies to increase cytotoxic immune responses in conjunction with anthocyanin treatment, potentially through combination with immune checkpoint inhibitors or adoptive cell therapies.

Another notable finding is the effect of anthocyanins on the tumor vasculature. The observed reduction in CD31^+^ endothelial cells following treatment suggests that anthocyanins may exert antiangiogenic effects, limiting the blood supply to the tumor and potentially enhancing immune infiltration. The dual role of anthocyanins in both immune modulation and vasculature regulation presents an intriguing therapeutic angle, particularly in solid tumors where angiogenesis plays a critical role in disease progression and metastasis.

Beyond their biological effects, an important consideration for the clinical translation of anthocyanins is the feasibility of large-scale production. The extraction and purification processes used in this study are cost-effective and scalable, making them suitable for clinical applications. However, while intratumoral administration demonstrated significant antitumor efficacy, oral delivery remains the preferred route in clinical oncology due to its practicality and patient compliance. The main challenge with oral administration is achieving sufficient bioavailability and tumor-targeted delivery. Therefore, further research should focus on developing advanced formulation strategies, such as nanoparticle-based carriers or prodrug approaches, to improve anthocyanin stability, systemic absorption, and tumor specificity. These advancements will be crucial in bridging the gap between local and systemic administration and maximizing the therapeutic potential of *Hibiscus sabdariffa* anthocyanins.

Despite these promising findings, the limitations of this study must be acknowledged. The incomplete tumor eradication and lack of robust T-cell recruitment following anthocyanin treatment suggest that these compounds may be more effective in combination with other therapies than stand-alone agents are. Additionally, the complex adaptive responses observed via gene expression analysis indicate that cancer cells may activate multiple survival pathways to resist treatment, necessitating a more targeted approach to disrupt these compensatory mechanisms.

In conclusion, this study highlights the potential of *H. Sabdariffa*-derived Dp3-sam and Cn3-sam anthocyanins as adjunctive therapies in cancer treatment. The ability of these compounds to modulate the tumor microenvironment, enhance innate immune cell recruitment, and inhibit tumor growth makes them promising candidates for combination with conventional therapies such as chemotherapy. Further research is needed to optimize their use in combination regimens, particularly in enhancing cytotoxic immune responses and preventing tumor regrowth. Ultimately, understanding the molecular mechanisms underlying their antitumor effects will be key to developing more effective anthocyanin-based therapies.

## Data Availability

The datasets presented in this study can be found in online repositories. The names of the repository/repositories and accession number(s) can be found in the article/Supplementary Material.
